# Exploring the Potential for Smartphone-Based Reflection to Support Socioemotional Competence Development in Student Nurses’ Clinical Placement: Mixed Methods Field Trial

**DOI:** 10.2196/91083

**Published:** 2026-08-05

**Authors:** Ivo Bril-Broers, Nick Degens, Joke Fleer

**Affiliations:** 1 Department of Health Sciences, Section Health Psychology, University of Groningen, A. Deusinglaan 1, Groningen, The Netherlands, 31 50 595 3439; 2 Research Group Human-Centered Technologies, Cluster Arts, Design & Built Environment, Hanze University of Applied Sciences, Groningen, The Netherlands; 3 Research Group (Dis)Connected Technology & Creativity, University of the Arts Utrecht, Utrecht, The Netherlands

**Keywords:** mobile app, smartphone, education, mixed methods, field trial, technology enhanced learning, nursing students, clinical placement, nurse, design research, social-emotional competence, soft skills, clinical practice, nursing education, reflection

## Abstract

**Background:**

Socioemotional competencies (SEC) are essential for nurses to provide quality care and to maintain their own health. Historically underrepresented, nursing programs are changing their curricula to incorporate SEC development more strongly. Although it is recommended to practice SEC during clinical placement, studies on how to support nursing students in developing these competencies are lacking.

**Objective:**

Our study explores second-year nursing students’ perceived and actual use of a reflection tool during their first 20-week clinical placement. Designed to support SEC development, the study focuses on how students incorporate the application in their learning process for SEC development.

**Methods:**

An embedded mixed method study was conducted with 15 students from a nursing program in the Netherlands. Qualitative surveys and semistructured interviews were used to capture students’ experiences with the application both during and at the end of the clinical placement. Log data from the application provided insight into how the app was used. The mixed methods data were analyzed using warranted assertion analysis.

**Results:**

The app helped students relate learning goals to clinical experiences, identify learning opportunities, and experience a sense of growth. Features that removed practical barriers to reflecting were experienced as most beneficial. Although SEC-focused use was limited (n=7), involved students used the app over a longer period of time and reported greater benefits. Nevertheless, 13 students stopped using the app halfway through their clinical placement, as their attitude shifted from learning to working.

**Conclusions:**

This study gives a rich description of how nursing students used a reflection app to structure their learning process during clinical placement. It reveals that smartphone-based reflection supports students in clinical placement learning. Specifically, the app lowers barriers to engaging in structural reflection and decreases the mental burden of self-regulated learning. However, more guidance and a multimodal intervention approach seem necessary for SEC development. Further research into how to account for students’ shifting attitudes toward learning is needed.

## Introduction

### Background

Socioemotional competencies (SEC) play a large role in the day-to-day work of a nurse, both to provide quality care as well as to maintain their own well-being [1-5]. As our understanding of the importance of these nursing competencies grows, so too does the interest in how to effectively teach [3,6-8] and assess [1,9] SEC in nursing programs.

A recent systematic review underlines the importance of developing SEC during clinical practice [10], showing nursing students are unprepared for the social dynamics of the workplace otherwise. Traditionally, however, the focus of clinical placement has been on improving technical skills [11-14]. Concurrently, nontechnical skills such as SEC are expected to be learned through a social and more implicit learning process [15-17]. The review’s findings suggest supporting SEC development within the clinical learning environment requires specific didactical attention. Yet, there is little research on what clinical placement needs or how to implement it there.

Research findings indicate that SEC require nursing programs to adopt active and innovative learning methodologies in their curricula [8,18,19]. Studies show that when reflection is used as an active learning methodology, SEC development is supported [20-23]. Reflection helps students to process their clinical experiences and learn from them [23]. Specific to SEC, reflection can support the development of interprofessional communication and collaboration skills [20,21], as well as a better understanding of oneself and the workplace experience [22-24]. Thus, using reflection specifically to make the implicit learning process of SEC more explicit warrants further exploration.

Over the past decades, studies on reflection within clinical placement have explored the potential of smartphone technology for its ease of use and ability to support students’ development when workplace coaching is limited [11,14,25,26]. When it comes to clinical skills, research shows this to be an effective approach to improve learning during clinical placement [26-28]. Moreover, many studies show nursing students, educators, and preceptors favor digital apps over paper-based methods of reflection [13,27-31]. Reasons for this preference include greater flexibility, a more convenient method of managing one’s reflections, and better visualization of one’s progress [29]. Although smartphone-based reflection can support clinical skill development, there is little research on how such apps can support SEC development.

This study aims to address this gap and explore the potential of smartphone-based reflection for SEC development during clinical placement. The study is part of a larger project in which we developed a reflection app called Nurse-IT in co-creation with nursing students and educators. The app aims to help students structurally reflect on their clinical experiences for SEC development purposes.

In the development process of the app, design guidelines from both theory and practice have been translated to concrete design features (eg, daily and weekly reflections). Given the lack of applied research on this topic, our study’s main focus is to explore the in situ impact of a SEC-focused reflection app on students’ learning processes. By focusing on students’ experiences with the app from a learning process perspective, we better understand not just the app’s impact, but also how it interacts with the learning environment in which it is introduced. Doing so provides a more nuanced and practical understanding of the developed app and the underpinning design guidelines. This leads us to the following design research question: *How do the app’s features impact nursing students’ socioemotional competency development during clinical placement?*

To answer this question, we conduct an embedded mixed methods field trial of the Nurse-IT app. By gathering a diverse set of data during the field trial, we aim to get a detailed view on how nursing students incorporate the app’s structural reflection in their learning process. We also measure their perception and usage of the app throughout the clinical placement to better understand how usage develops over time.

### The App

#### Design Guidelines

Designing educational interventions requires incorporating and balancing both theory and practice. We began with an overarching learning process based on theory and clinical placement as a learning context. This was developed further into a prototype through co-creation. We briefly summarize the most salient design guidelines and the app itself here.

#### Theory

**Figure 1.**
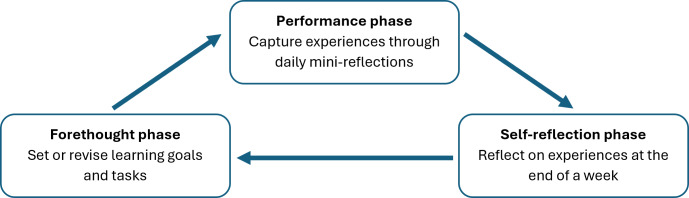
Self-regulated learning cycle which starts at the forethought phase, to then monitor oneself in the performance phase, and to evaluate oneself in the self-reflection phase after which the cycle begins anew.

The clinical learning experience focuses on learning-by-doing and real-life practice, with students encountering many social situations during a shift. Part of the app’s goal is to support students in capturing those moments to be able to reflect and learn from them, facilitating further experiential learning [32]. Moreover, the aim is to empower them to structurally and intentionally work on their SEC, resulting in a self-regulated learning cycle [33] (Figure 1). Finally, as an educational app is dependent on students using it consistently, its design needs to make the primary task (ie, reflection) easy and enjoyable. For this, we used the principles of persuasive systems design [34].

#### Practice

Nursing students and educators of a nursing program in the Netherlands were involved to make sure that the app’s design and user experience align with their needs [11,14,30,35]. Although the overarching learning process was determined beforehand, the co-creative process was necessary to translate it to a design that corresponds with the reality of doing a clinical placement. One outcome of the entirety of the co-creative process was the formulation of five design guidelines:

1. Provide structure, but allow for flexibility: The app needs to scaffold goal-setting and reflection by providing preset questions [36]. Those questions should allow students to express themselves in a way that suits their needs.
2. Low time-investment when capturing experiences: The performance phase requires students to structurally capture their experiences. Given the high workload of clinical placement, capturing those experiences should be quick and easy to do. Even if that means having a low level of reflection.
3. Looking back, but also planning ahead: The app should facilitate students in planning follow-up actions based on what they learned from their reflections.
4. Help me see, monitor, and appreciate my growth: The app needs to summarize and visualize the student’s growth toward their learning goals. In doing so, the students should feel in control of their learning process [35].
5. No one has access but me, but I can share things if I want: To further underline student autonomy and learner agency, the app’s contents should only be visible to the student [37,38]. Nothing should be assessed unless the student wants to share something themself.

#### Features of the App

##### Forethought Phase: Learning Goals and Tasks

Clinical placement requires students to reach specific learning outcomes. Goal-setting helps structure this learning process and represents the forethought phase of self-regulated learning. Moreover, setting goals allows the students to monitor their progress and stay aware of the goals they are working on [39]. Consequently, learning goals are a core feature of the app: students formulate their own or use the curriculum’s competencies. They then add a target grade they want to work toward and determine the period in which they want to work on the learning goal (guideline 3).

Another aspect of the forethought phase is planning. Setting a goal is one thing, but breaking it down into smaller, more achievable parts (ie, proximal goals) helps focus students’ efforts toward achieving the goal and raises self-efficacy [39]. This also ties into experiential learning’s phase of active experimentation. The app facilitates this by allowing students to add tasks to their learning goals (guideline 1).

**Figure 2.**
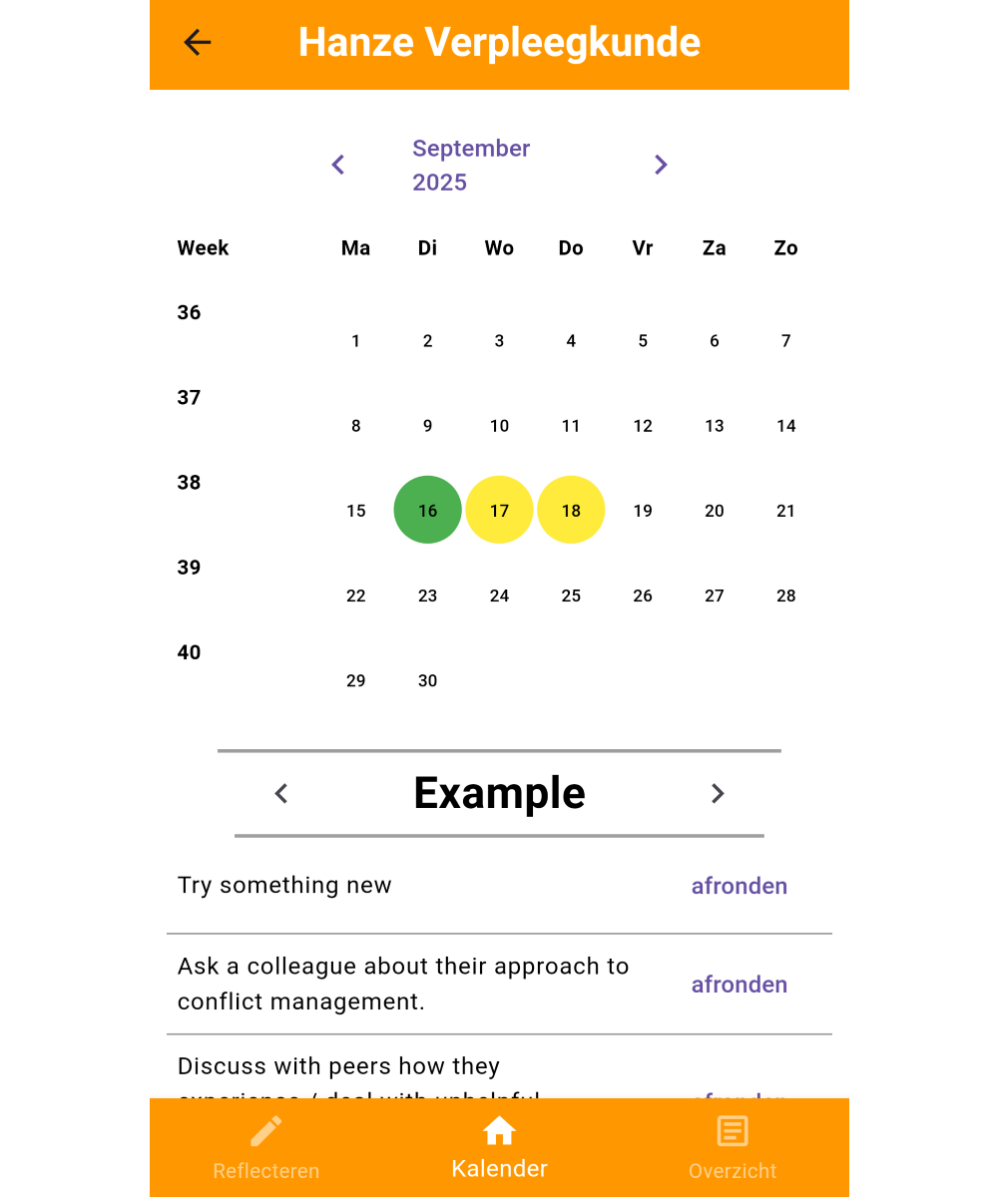
Home screen of the app. The calendar shows a month with 3 completed daily reflections, each represented by a colored dot. The word ‘Example’ represents a learning goal, with the lines of text underneath each representing an individual learning task.

To further help students be aware of their learning goals and respective tasks, they are placed on the home screen of the app (Figure 2). Here they can navigate between learning goals and check off tasks (guideline 4).

##### Performance Phase: Daily Reflections

Daily reflections represent the performance phase of self-regulated learning. The main goal of daily reflections is to have the student self-monitor and evaluate after each shift (guideline 4). To ensure students are willing to do so, we favor a low time investment over achieving deeper reflection (guideline 2) [34]. In doing so, we aim to achieve two things: (1) students record more situations and build up a reservoir of experiences which they can reflect on, and (2) students engage in a frequent and structured way of reflection-on-action and thus have better awareness of their progress (ie, self-monitoring) [33,39].

The daily reflection’s design reflects these goals by using features that provide immediate low-level insights and help summarize an experience from both a descriptive (eg, title and tags) and an emotional level (eg, emojis). In terms of evaluation, the student (1) grades themselves on the learning goal they are writing the reflection for, (2) can write a *tip* for themselves, something they could do differently next time, and (3) can write a *top* (ie, a positive comment) about what the user felt went well. Finally, the student can use a text field to write about the shift in whichever way they want (called “free writing”). It is to be noted that we only require the student to give a grade to their shift; all other features are optional. This way we follow the design guideline of providing structure but allowing for flexibility (guideline 1).

##### Self-Reflection Phase: Weekly Reflections

With the daily reflections covering the performance phase, the weekly reflections represent the self-reflection phase and the revisiting of the forethought phase of self-regulated learning. Consequently, the main goal of the weekly reflections is twofold: (1) to achieve deep reflection and use different experiences gained through daily reflections, and (2) to review one’s progress and make plans for the week to come (guideline 3).

This is translated into the app through a three-step process, aligning with experiential learning principles (and guideline 1):

1. A student looks back at the week and describes what they are proud of and what they found difficult.
2. They choose one of the 2 and reflect on why they feel that way, what situation caused those feelings and how they dealt with the situation, what they learned from the experience, and how they would approach similar situations next time.
3. They review their learning goals and select those that relate to the week’s reflection. Learning goals and tasks can be marked as complete, edited, or newly created.

Ideally, a weekly reflection helps students identify when they are stuck in their learning process and need to reach out to others for help. As the reflection app keeps the students’ learning goals and reflections private to themselves, only the student can act on app-related insights. This, combined with findings of our co-creative process and earlier work that show students can have trouble identifying such situations [17], made us aim to create triggers for help-seeking behavior.

##### Learning Goal Overview

**Figure 3.**
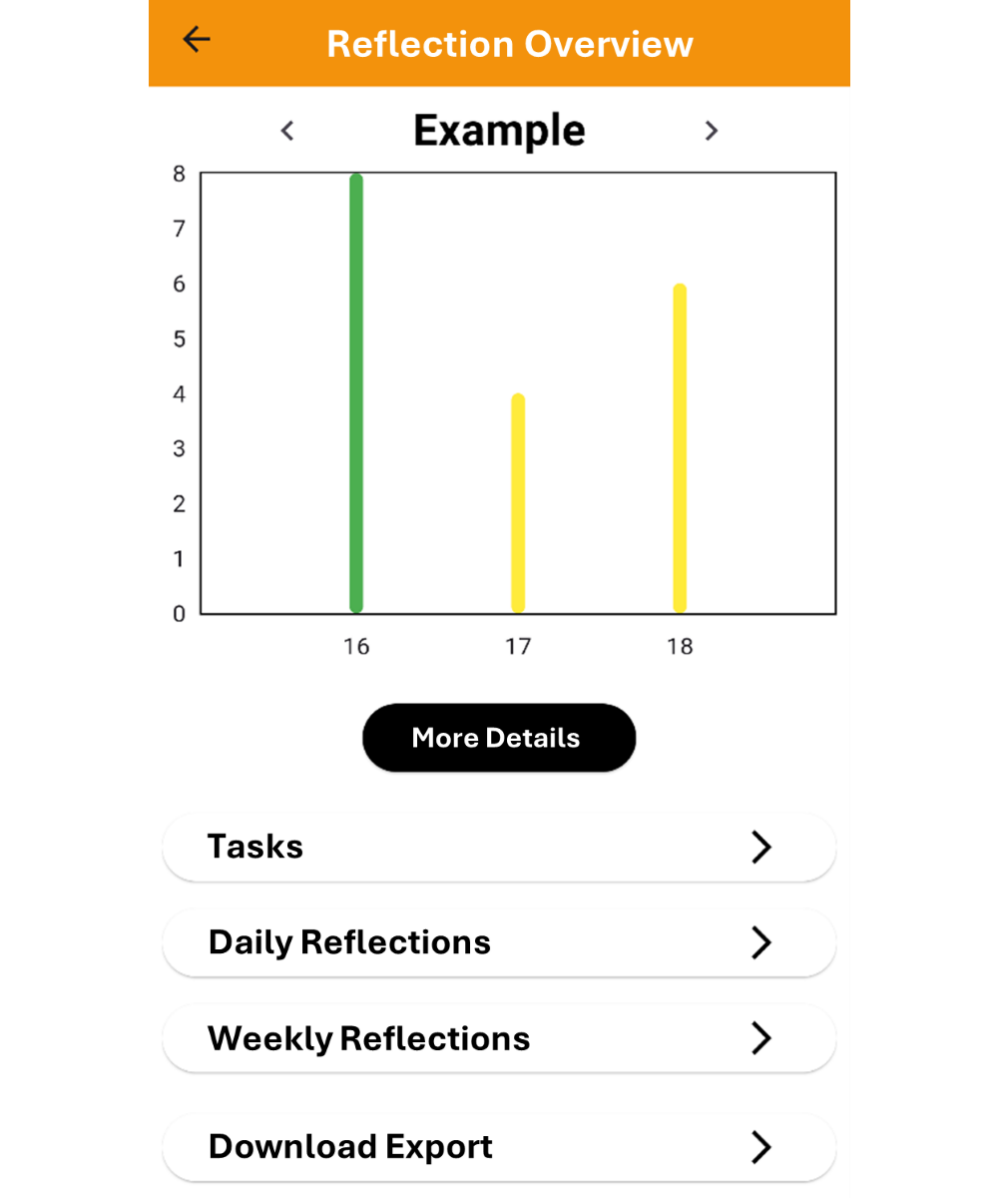
Overview of an example learning goal. The graph shows the grades of three daily reflections, the color indicating the grade they gave themselves (1‐3 red, 4‐6 yellow, 7‐10 green). The ‘tasks,’ ‘daily reflections,’ and “weekly reflections” buttons lead to their respective overview pages. Download export provides a spreadsheet version of the learning goal’s activities.

Students can review their active and completed learning goals through the overall learning goal overview screen. Here, they can also review their progress on a specific learning goal through the learning goal overview (guideline 4), which harbors all the related daily and weekly reflections, tasks, and visualizes the daily reflection grades over time (Figure 3). As per the design guideline concerning sharing data, this page also allows students to export their learning goals for documentation or coaching purposes (guideline 5).

**Figure 4.**
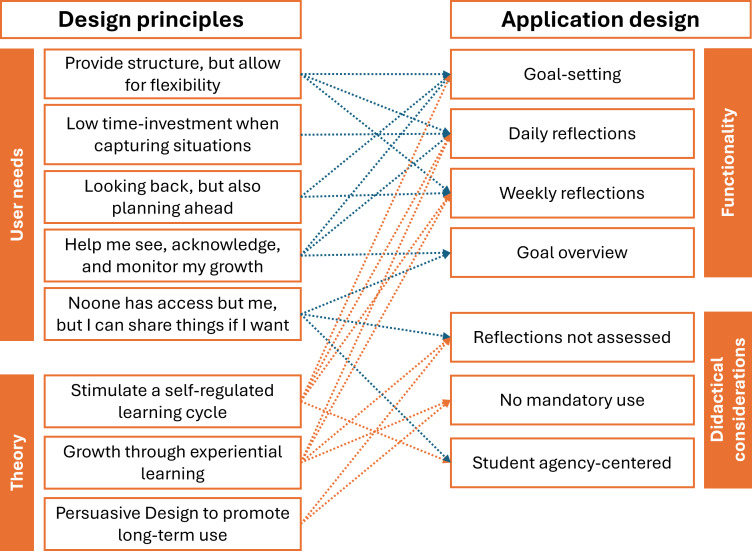
Overview of how the design guidelines are instantiated in the design of the app.

Having discussed the features of the app, Figure 4 summarizes their connection to the design guidelines.

## Methods

### Study Design

The study used an embedded mixed methods research approach within a pragmatist paradigm to capture the potential richness of a naturalistic field trial. The goal of mixed methods research is to integrate knowledge of several methods, both quantitative and qualitative, allowing meta-inferences to be drawn [40]. These meta-inferences aim to explain phenomena in a way that exceeds the sum of a study’s parts (ie, each individual method). Thus, describing the selection of methods as well as the purpose and timing of integrating results in mixed methods studies is important.

For this study, reflection contents, open-ended surveys, and interviews give a qualitative perspective on how students used the tool, how that usage developed over time, and a rich description of their context of use. Embedded within this qualitative study are log data. These give a quantitative perspective on how participants engaged with the tool. By combining perspectives, our understanding is informed both from the actual usage of the tool, as well as the participants’ context-rich experiences with the tool. Together, both strands help illuminate the app’s role within the student’s learning process: which parts of the app are used, what needs do they meet, and why?

**Figure 5.**
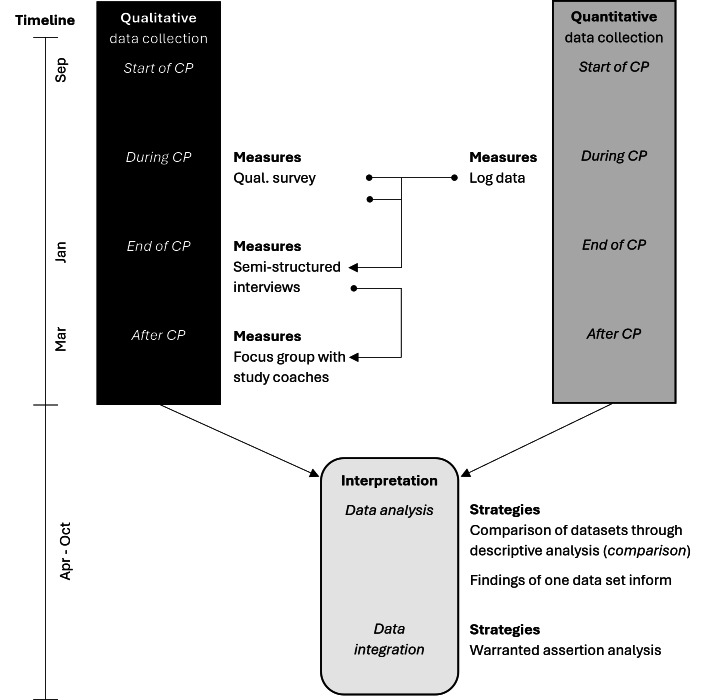
Visualization of the embedded mixed methods study design. A predominantly qualitative study embedded with a quantitative method to answer the research question from both a perceived and actual use of the app. CP: clinical placement.

It is recommended to visualize the mixed methods design in a diagram to condense its complexity in an explainable manner [41]. In Figure 5, we provide a chronological, vertical diagram of the above-mentioned methods. Arrows between methods highlight how insights from one method informed the data collection of another (ie, *building* [40]). Data interpretation (ie, analysis and integration) is simplified in this diagram and will be discussed in the Data Interpretation section.

### Measures

#### Actual Use of the App

##### Log Data

In addition to participants’ own assessment of their engagement, log data were recorded of in-app activity to capture actual use of the tool. Participants’ sessions, the creation of a learning goal or daily reflection, and the usage of tags were logged. Additionally, participants’ learning goals, tasks, daily reflections, and weekly reflections were also stored in a database for both quantitative and qualitative analysis.

For the purposes of determining whether students were still active, in-app activity was monitored on a weekly basis. If students had been inactive for over 2 weeks, they were considered to have stopped using the app.

##### Reflection and Learning Goal Contents

Contents of the reflections and learning goals were included in the study to better understand how the features were used. This helps to further interpret the log data and possibly distinguish different patterns of use (eg, whether SEC were set as learning goals).

### Experience Using the App

#### Qualitative Survey

To understand participants’ experiences with the app, we developed a small survey with seven open-ended questions (refer to Multimedia Appendix 1 for the survey questions). These focused on the daily and weekly reflection features, as well as how students experienced the balance between time and the insights they gained from using the app. This survey was set to be distributed three times: the third, fifth, and seventh week of the clinical placement. The qualitative survey helped capture the early development of the student’s engagement with the tool. Due to a severe technical issue further described in the limitations section, only the first 2 surveys were administered. The first on September 19, 2024, the second on October 10, 2024.

#### Posttrial Semistructured Interview

Semistructured interviews were conducted online between December 18, 2024, and January 22, 2025. Our goal was to understand the role of the app in participants’ learning process during the clinical placement (refer to Multimedia Appendix 2 for the interview schema). Participants were reminded to give their open and honest opinion about the app. As other measures already provided a clearer picture of the actual use and, to an extent, what it was used for, the interviews focused on:

1. Participants’ context of learning
  - The clinical placement as learning environment
  - Their perspective on the clinical placement (eg, expectations and motivation)
2. Participants’ learning process
  - What skills did they focus on, and how did they approach learning itself?
  - How structured and self-regulated was their process?
3. How did those related to the app
  - How did the context of learning influence app use?
  - What was the role of the app in the participants’ learning process?

To be able to compare learning processes of active users and students who did not, or hardly, use the app, students who dropped out during the trial were also invited for this interview. For these cases, the last topic focused on why the app was not, or hardly, used.

#### Posttrial Focus Group With Study Coaches

After the interviews, a coach-perspective was required to verify whether certain observations were commonly seen in the broader student population, or just our sample. Of our invitation to the entire coaching team of this specific clinical placement, 4 coaches agreed to participate. All gave verbal consent (refer to Multimedia Appendix 3 for the topic list). The focus group was conducted on March 18, 2025, took approximately 45 minutes, and focused on (1) students’ changing attitude toward learning during the clinical placement, (2) differences between identified groups of students, and (3) level of challenge of the second-year clinical placement.

### Data Interpretation

The goal of mixed methods research is to find meta-inferences through the integration of qualitative and quantitative findings. One strategy to do so is through *warranted assertion analysis* [42], which consists of four steps:

1. Organizing the raw data of each individual data set
2. These data sets are then read as a whole, repeatedly, to come to warranted assertions (ie, meta-inferences)
3. Evidence is collected for each warranted assertion
4. Warranted assertions are refined iteratively by looking for disconfirming evidence

This inductive approach toward mixed methods analysis was chosen as each of our measures shed light on a different aspect of the research question. Although some revealed more than others, they needed to be treated equally valuable for meta-inferences to be drawn. Put in Smith’s [42] words:

*That is, whether data happen to be in the form of words or in the form of numbers should not materially affect the process of constructing meaning from them. Both forms of data are symbolic and neither is inherently “harder” or “softer” than the other*.

We will first report our initial analysis on a measure-to-measure basis. We then describe how we integrated qualitative and quantitative data to reach warranted assertions. The findings section will describe the findings of each measure, after which we discuss our warranted assertions in the Discussion section.

### Data Analysis Per Measure

Log data were analyzed quantitatively through descriptive measures (medians, means, and SDs when applicable) to see students’ actual usage of the app. Students’ activity was quantified through (1) the number of days between first and last use; (2) the number of sessions within that time period; (3) the number of learning goals, daily reflections, and weekly reflections created; and (4) the number of tags and emoticons used (both total and average per session).

Learning goal contents were analyzed to see what topics students used and whether they were SEC or clinical skill-related. This was done by (1) creating an overview of all learning goals, categorized per participant; (2) comparing learning goal phrasing; and (3) cross-referencing it with the corresponding daily reflections to see how the learning goal was used in practice. An additional category (introductory period) was later added to refine the categorization.

During this round, reflection contents were only analyzed to get a first impression of how students used both the daily and weekly reflections. This was done by (1) creating an overview of an individual participant’s daily and weekly reflections, (2) reading the reflections, (3) summarizing the participant’s approach to reflecting (what fields were used and overall focus of the reflection).

The qualitative survey data were analyzed for perceived ease of use and utility of the daily and weekly reflections. Per question, responses were analyzed using content analysis. These results were then compared between the 2 questionnaires to analyze potential differences.

The interview schema was used as a lens to structure the semistructured interview analysis. Each transcript was analyzed separately to describe each student’s individual learning process and how they incorporated the app.

### Data Integration

The data integration process consisted of repeatedly engaging with the results of the data analyses and the raw data. Performing the data analysis and comparing the results between measures was the first step in identifying potential warranted assertions. Then, using the semistructured interview findings as a starting point, these potential warranted assertions were refined, and new ones were found by *importing* [40] the other methods’ findings one by one. For example, importing the reflection and learning goal contents helped us understand patterns of use in relation to students’ approaches to learning and their learning environment.

Throughout this process, the integration shifted between a student-specific focus and an overarching one. Doing so allowed us to scrutinize the warranted assertions for disconfirming evidence (ie, if a student’s learning process did not fit an assertion), resulting in a more nuanced analysis.

### Recruitment

#### Procedure

The sample consisted of Dutch nursing students of the Hanze University of Applied Sciences in the Netherlands. Inclusion criteria were students attending their second year and who were going on their first 20-week-long clinical placement.

Students were recruited using convenience sampling through the following means:

First, a call (and several reminders) was posted on the study program’s educational intranet, specifically the clinical placement page where students of our sample were enrolled. It featured a brief description of what the field trial entailed and its goals, a link to sign up, and a reference to the information letter.

Second, an information letter was shared on the same educational intranet page as the call. This letter included an explanation of the goals of the study, what participation entailed, how data would be managed, the potential benefits and risks of participation, and how students could drop out during the study.

Third, an information session was held by the first author (IBB) following a general information session about the clinical placement. In this session, the basic features of the app, the setup of the field trial (eg, what data would be collected and when), and how to sign up were explained.

Finally, the start-up session of the field trial was deliberately planned to follow the main start-up of the clinical placement organized by the study program. This allowed students who had missed the call before the summer break to still sign up. Additionally, as this was the start-up to the curriculum’s first clinical placement, this also guaranteed our participants met our inclusion criteria.

The start-up session was given by the first author (IBB), someone the students had no previous relation with and who does not fulfill a role within the curriculum. The session included a reiteration of the study’s goals and data collection, an explanation of the features of the app, how students could use it to showcase course-related competencies, and a hands-on support session to help students get the app running on their phones.

#### Participants

In total, 34 participants signed up for the study, but only 23 participants started. Moreover, 5 were excluded during the screening process as they were 3rd- or 4th-year students, respectively, doing their second and third clinical placement. In total, 6 participants dropped out at the start-up session.

**Figure 6.**
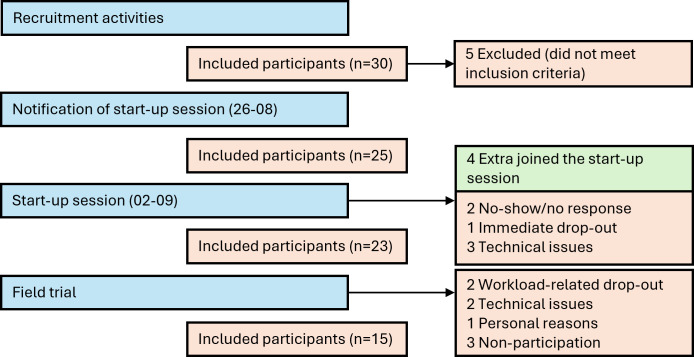
An overview of the recruitment process for the field trial.

In the first 3 weeks of the trial, we had 5 more dropouts: 2 due to the workload of the clinical placement, 2 due to unsolvable technical difficulties, and 1 due to personal reasons. Moreover, it became clear near the end of our trial that 3 participants had hardly been active in the app, had not filled in any surveys, and did not respond to our interview request. This brings the number of active participants down to 15 (refer to Multimedia Appendix 4 for the demographic data). Figure 6 gives an overview of the process.

### Ethical Considerations

A risk to study participation was the added workload of maintaining learning goals and reflections in the app, with no guarantee that this work would aid in passing the clinical placement itself. As the clinical placement is partially graded based on personal growth with regard to several learning outcomes (including 2 personal learning goals), we arranged with the clinical placement coordinator that the activity within the app could be used as evidence for such growth. In doing so, we aimed to lower the risk of participation and ensured that students gained something by participating. We prepared alternative material in case technical errors would occur (same format and questions but in separate Word files).

Ethical approval was obtained from the Hanze Ethical Advisory Committee before the commencement of this study (heac.T2024.032).

Informed consent was obtained at the end of the start-up session. Data were stored on the cloud storage of the researchers’ university. As multiple data gathering tools were used, participant data were pseudonymized to allow for data comparison and importation. The translation key was stored in a separate cloud storage which only the lead researcher had access to. The data will be deleted 5 years after the project’s completion. Due to low response rates to the first questionnaire, a small financial compensation was added to the study. The reasoning behind this choice is discussed further in the Limitations section.

## Results

### Actual Use of the App

#### Log Data

All students used learning goals, with the second most used feature being the daily reflections (14/15, 93.3%). Weekly reflections were barely used (8/15, 53.3%), of which only 1 used it multiple times. Emojis were used by a smaller group (6/15, 40%), with half of those users using at least one emoji per daily reflection. Tags were more broadly used (12/15, 80%; mean 1.76, SD 1.63), but intensity of use varied. Moreover, 3 students used tags and emojis frequently, with on average more than 3 tags and 1 emoji per daily reflection.

App usage varied strongly (mean 46.7, SD 20 d), with 3 outliers who used the app for more than 2 months (not the same 3 as the tag- and emoji-users). Most students used the app for 6 weeks in the first half of their clinical placement (9/15, 60%). No one completed goals, with overall goal use showing 3 patterns: creating all learning goals at the start (4/15, 26.7%), creating learning goals throughout clinical placement (6/15, 40%), or only creating a few at the start (5/15, 33.3%). Due to logging issues, we were unable to accurately determine session lengths.

#### Reflection and Learning Goal Contents

Qualitatively, learning goal contents show three categories: focusing on clinical skills, the introductory period, and SEC goals (refer to Table 1 for an overview). Only half of the students set SEC goals (n=7), whereas nearly all of them set clinical skill (n=13) and introductory period (n=12) goals.

**Table 1.** An overview of the 3 identified goal categories, how many students used them, and illustrative examples.

Goal categories	n	Illustrative goals
Clinical skills	13	“improving practical skills” [Participant 264] “independently doing the handover to the next shift” [Participant 451]
Introductory period	12	“getting to know the patients” [Participant 269] “getting up to speed with protocols” [Participant 380]
Socioemotional competences	7	“setting boundaries” [Participant 960] “clearly and empathically react to and give feedback” [Participant 528]

Reflection contents for clinical skill-focused goals included summaries of executed procedures and self-assessments of what went well or needs to be improved. Similarly, introductory period-focused reflections mainly consisted of self-assessments. SEC-focused reflections were often more elaborate, with a stronger focus on emotions and actions. Examples are illustrated in Table 2.

**Table 2.** An overview of the three goal categories, their predominant characteristics, and example quotes.

Goal categories and reflection content	Illustrative quotes
Clinical skills	
Summaries	“I washed people only when asked to do so. I emptied catheters. I helped people go to the toilet. I gave medication to a few people, but it was busy so my colleague did most of it herself. I restocked cupboards with towels myself.” [Participant 664]
Self-assessment	“Next time, I should ask if I can do something, like wash their back or face.” [Participant 269]
Introductory period	
Self-assessment	”I notice that I am starting to feel comfortable with the various clients and that I can talk with colleagues.” [Participant 309]
Socioemotional competences	
Focus on emotions and actions.	“I had to do the caretaking tasks for a lady, after which she gave me one task after another while I was still working on the first one. She also said this in a very commanding tone. After this, I stopped and told her that I can only do one thing at a time and that I am doing my best. It frustrated me and made me feel uncomfortable. After that, she apologized and was much calmer. The colleague who was in the resident’s living room also said that I had understood the situation well and responded appropriately. This made me feel very good and confident.” [Participant 202]

The tip- and top-fields were used often and similarly across goals. The tip-field often being a small, future-action-oriented reflection (eg, “project more confidence” [Participant 402]) related to the described experience, and the top being a reflection on what went well (eg, “indicated more clearly when I didn’t understand something” [Participant 246]).

### Experience Using the App

#### Qualitative Surveys

##### First Survey

The 9 participants who filled out the first version also filled in the second. Moreover, 6 other participants only filled in the second version. Although this limits our potential to compare changes within individual participants, there is enough data to look at more overarching trends.

In line with the log data, results of the first questionnaire show participants appreciate the daily reflection feature (questions 1, 1a, and 3). Specifically, students mention its ease of use:

*It brings me a lot. And the questions make it easy, so it takes less time.*

[Participant 907]

Additionally, they like how the formatted questions provide focus (eg, tip or top):

*That you can give tips and tops so that you can clearly see what you are doing right and wrong.*

[Participant 246]

Finally, students appreciate that these reflections are brief but still provide small, valuable insights:

*It helps me process things by writing down what has happened, while typing, I have sometimes come to realize things.*

[Participant 202]

Some participants remark that it is hard to remind themselves to use the app after a shift, asking for a reminder feature (question 3):

*It might be nice to have the option to receive notifications. I notice that I quickly forget about it myself*.

[Participant 309]

The few students who have used the weekly reflections were positive (question 2 and 2a):

*Quite a lot, actually! It’s nice to have a slightly clearer structure and to answer the questions*.

[Participant 926]

However, the majority of the students mention they still have to try out the weekly reflection. Generally, the first questionnaire shows that about a third of the participants engaged little with the app.

##### Second Survey

The second survey also shows participants are positive about the daily-reflection feature (questions 1 and 1a). They describe the added value more clearly:

*I reflect on things I learned that day and things that are quite special, which I would not have thought about without the questions.*

[Participant 202]

The week reflections remain underused, even though participants say they are interested. Those that have used it appreciate its reflective format (questions 2 and 2a).

*Especially step 2 in the weekly reflection (the analysis). By asking follow-up questions about the questions from step 1, you really start to think deeply about why you feel a certain way. The questions also make me feel thankful!*

[Participant 451]

About half of the participants state they get value from the overview page of their learning goals. The way the other half uses the app is not supported by the app’s design (question 4).

*Personally, I don’t pay much attention to the charts, tips, and tops. This is because I mainly use free writing*.

[Participant 163]

*I hadn’t really reflected on learning objectives yet; I had mainly written reflections about the days themselves*.

[Participant 363]

The second survey shows participants appreciate the incremental, iterative growth of the daily reflection- and learning goal-features. This supports them to be more mindful of their learning process and goals (question 3):

*This way, I remember much better what I’ve done, and I often find that I’ve done more than I think.*

[Participant 664]

### Posttrial Semistructured Interviews and Coach Focus Group

In total, 13 participants responded to our call for a posttrial interview, 2 of whom had not used the tool but had enrolled for the study at the start. We specifically included nonusers to better understand the perspective of students who had the opportunity to use the app but chose not to engage with it.

### Fading App Usage

Nearly all app-using participants remarked that usage faded over time. A commonality among the reasons given was the transition students experienced from an initially challenging environment to one in which they experienced more confidence in their capabilities and were less in need of ways to structure their experiences. This decreasing urgency is mirrored by the decrease of in-app activity as seen in the log data.

Combining students’ accounts of this transition with the reflection contents and learning goal categories, we see one distinct usage pattern which affects this (refer to Table 3 for a joint display). The pattern consists of students who used the app predominantly for clinical skill learning goals by logging performed procedures. They describe the transition as moving from a “learner” to a “worker” attitude or “just doing the work.”

**Table 3.** Joint display showing students’ pattern of use compared with their learning goals per category and an illustrative quote of their reason for quitting the app (this table only includes students who were interviewed).

Pattern of use and participant	Learning goals	Illustrative quote for quitting the app
Clinical skill		
246	CS^a^=2 IP^b^=2 SEC^c^=0	“But after a month, I had a pretty good handle on it, and now it’s just work, work, work.”
269	CS=6 IP=1 SEC=0	“Yes, I’ve become so ingrained in that department that it no longer feels like I can really learn much more, so to speak.”
451	CS=1 IP=1 SEC=0	“Because I was getting more into the flow of work, I was, like, less focused on the school’s learning objectives. It became more like work instead of [learning].”
664	CS=1 IP=3 SEC=0	“It’s all pretty much the same. (.) I didn’t really do much with it, so I noticed, like, that as the internship went on, I’d start thinking, well, should I write something down again this afternoon, or not? Or should I? Or what am I going to do? And then decided I didn’t feel like it.”
960	CS=5 IP=0 SEC=5	“Well, it’s because I’m really focusing on the new part of my internship. That I’m putting a lot of interest and energy into learning what they do there. So then that extra part about how I should work on myself, I kind of disregarded it.”
Socioemotional competences or journaling		
163	CS=0 IP=1 SEC=0	“And at some point you think, if it’s been two or three days, ‘Oh well, that’s not fresh in my memory anymore, is it?’ I’ll just make sure to do it another time. But then, that ‘another time’ sometimes never came.”
363	CS=0 IP=2 SEC=1	Severe technical issue
402	CS=1 IP=1 SEC=1	Severe technical issue
528	CS=4 IP=5 SEC=5	Severe technical issue
Other		
309	CS=1 IP=3 SEC=0	“I quickly had this intuitive sense of how things were going, so I didn’t feel like I needed it.”
907	CS=0 IP=1 SEC=0	“I don’t know, I just didn’t manage to find my way around in the app all the time”

a CS: clinical skill.

b IP: introductory period.

c SEC: socioemotional competences.

*Yes, I don’t think about it as much. That’s a shame, of course, because you want to keep pushing forward and keep learning. But over the past few weeks, I’ve noticed that I’m just working along with the team. I’m thinking less about like, what went well and what could go better*.

[Participant 451]

For some of these students, this shift in attitude coincided with a sense of having learned all there is to learn for this clinical placement.

*Well, I’m trying to challenge myself to work in other departments to see something different. But basically, yes, there are so few nursing procedures that when there were a lot, I could set a learning goal for each one. Yes, I’ve become so ingrained in that department that it no longer feels like I can really learn much more, so to speak*.

[Participant 269]

Most students who used the app for SEC learning goals or as a more personal journal of their learning experience quit due to the severe technical issue. For them, the app provided a consistent place for experiencing a sense of growth and peace of mind, and kept them from forgetting their experiences.

*Yeah, maybe it’s a little weird, but like it gives me some peace of mind or something. Because sometimes you’re in the middle of an internship day and think to yourself: why am I doing all of this anyway? And when you’re busy in the app, for me it brought a sense of, oh, right, I see now, I* have *been working on this after all, and I* have *grown a bit. Or at least stayed consistent with it, so it did give me some peace of mind, like, oh, all of this does have a purpose*.

[Participant 528]

*But in the end, it did provide an overview of everything you’ve learned. Because I think if you didn’t use the app or didn’t think about it every day, you’d end up trying to remember everything later on. Like, oh yeah, I think I learned this too. But if you really know what you did each day… Because at the end it all just becomes one big blur, so to speak, now you really know a bit about when you learned what*.

[Participant 402]

### Consequences of Fading App Usage

For all students, no longer using the app meant their conscious learning shifted to unconscious and, for some, more passive learning. The coaches also remarked on this:

*They learn a lot in the second half, but they are less aware of it because they are also more focused on working.*

Coaches acknowledged the transition being a common occurrence during clinical placement. Coaches mentioned that a low level of detail in a student’s plan of approach, written at the start of the clinical placement, often leads to this passive attitude. Other reasons came down to:

1. Whether the working environment fostered students’ learning as opposed to treating them as workload-relief
2. The desire of the individual student to continuously develop their SEC, as the first clinical placement’s bar is low in that regard
3. Whether students proactively sought opportunities and activities to learn from, as opposed to a more reactive or passive stance.

In line with the coaches’ observation, most participants remarked that no longer using the app also resulted in less awareness of their personal and professional growth.

*Yes, well, that was indeed the case. In the beginning, I used it a lot, and then the halfway evaluation came, and then I noticed that, yes, at least. In the beginning, I had a clear picture, but those last 2, 3, 4 weeks. It was just like I was working. As if it was just normal for me to do certain things. And only then I thought, yes, some things are actually not normal at all for us to already do*.

[Participant 163]

### Other Avenues for Reflection

The 2 students who had not used the app seemed to already have a structured learning approach: meaningful situations were noted in a physical journal and reflected upon. Additionally, both could identify and act upon relevant “learning moments” during their shift. This indicates that students who can self-regulate their learning might have less need for this app.

Other students shifted their reflective needs to colleagues or peers, depending on the clinical placement’s own learning environment. Like the app, colleagues in a learning-inducive environment could provide students with insight into their learning and progress. However, those discussions rarely focused on SEC.

Due to the severe technical issue, some students chose to use our nonapp alternative, albeit only briefly. The main reasons for students to prefer the app came down to (1) a clearer connection between learning goals and daily experiences, (2) a stronger sense of achievement upon seeing their progress in the app, and (3) the ease of use of a predefined format for reflection.

## Discussion

### Principal Findings

This study explored how and why nursing students used the Nurse-IT reflection app’s features to structure their learning process for developing SEC during clinical placement. Using an embedded mixed methods approach and a naturalistic setting, we were able to capture a detailed and realistic picture of how the app was used and how this usage changed during the clinical placement. We will first discuss how students used the app to structure their learning. We then highlight how that pattern of use differed from our intended use and what we can learn from it. Finally, we discuss students’ apparent changing needs during clinical placement and what that means for the design of such reflective applications.

### Role of the App in Student Learning

The overarching design goal of the app was to stimulate learner agency through scaffolding a self-regulated learning process (Figure 1). We modeled this learning process by defining *learning goals and tasks* (forethought phase), *daily reflections* (monitoring phase), and *weekly reflections* (self-reflection phase and forethought phase). This section discusses how and why parts of this process supported students in structuring their learning process.

Having the app facilitate a self-regulated learning process alleviated students of the burden of setting up such a process themselves. This is seen in how students valued the app for lowering their mental workload. For example, writing experiences down meant no longer having to remember them. Finally, students remarked how the preformatted questions made it easy for them to engage in goal-setting and reflection. These findings underline the importance of applying persuasive systems design principles when designing such tools [34]. Thus, taking away practical barriers makes engaging in self-regulated learning manageable for students.

Structural engagement with the app made students more aware of their learning process (eg, remembering learning goals during a shift). In turn, this awareness helped them identify learning opportunities and connect those to their learning goals. Other studies focusing on supporting reflection in nursing students show similar effects [12,28]. Survey and interview findings show students valued these effects the most. Overall, this highlights that structurally making one’s learning explicit enables students to act on it.

Moreover, and in line with other studies, students who structurally used the app also remarked how its various overviews helped them see and appreciate their growth [12,35]. This boosted confidence and motivated app usage. This effect was best seen in students who used the app as a reflective journal or to capture and evaluate experiences in relation to SEC-related learning goals. In contrast, students who only recorded clinical procedures appreciated their development, but noted it became repetitive and questioned its long-term value. Thus, making one’s learning explicit also motivates further action, especially when it is focused on SEC-related learning.

Arguably, this could be achieved through more affordable, physical notebooks. However, students who switched to our nonapp alternative said they missed how the app structured their learning process. For example, the link between learning goals, tasks, and daily reflections, and how the app summarized their growth. As a result, they stopped using the alternative, further underlining the benefits of smartphone-based support systems [29-31]. Thus, empowering students to have an overview and awareness of their learning process scaffolds a self-regulated learning process.

### Discrepancy Between Intended Use and Actual Use of the App

#### Overview

Our intended learning process included all features of the app. However, most students only used the learning goals (forethought phase) and daily reflections (monitoring phase). The weekly reflections were only used sporadically. Given the beneficial effects discussed in the previous section, this raises questions about the necessity of our intended learning process and the potential consequences of how students used the app. We will first discuss the assumptions behind our weekly reflection’s design, then relate those to the students’ favored pattern of use, the consequences of that use, and its implications for designing reflection apps for clinical placement in nursing programs.

#### Weekly Reflection Design Assumptions

We designed the daily reflection format to get to surface-level insights, with weekly reflections providing deeper reflection. Thus, although the daily reflections have some self-judgment (a part of the self-reflection phase), we believed the weekly reflections would be necessary for self-reflection.

Aside from depth of reflection, the weekly reflections also dictate a pace for the self-regulated learning cycle. Even though this was not technically enforced within the app, the term “weekly” indicates a pattern.

Finally, the weekly reflections consist of 2 steps—a guided reflection on an experience of the past week (self-reflection phase) and a check-up on one’s goals to prepare for the following week (forethought phase). With the latter, the weekly reflections represent the start of a new self-regulated learning cycle. Thus, we assumed students needed scaffolding for reviewing their goals, completing them, and setting new ones.

#### Students’ Pattern of Use and Its Consequences

A key aspect of how students used the app differently lies in how they approached their learning goals. Whereas we intended students to complete them and set new ones, the majority of students only set them at the start. Moreover, no one completed a learning goal. Instead, students set large learning goals, which they worked on throughout clinical placement. This negatively impacted the weekly reflections’ relevance.

Consequently, students’ self-regulated learning cycle took on a different form: students used the app to monitor their progress on these large goals. Their self-reflection and forethought phases aligned closely with the daily reflections’ features: how they are performing (eg, grade and top-field) and whether they need to adjust their approach (eg, tip-field and free writing). Additionally, the interview findings show self-regulated learning also took place *outside* the app (eg, reflecting with colleagues or peers). Overall, this indicates the weekly reflection was not needed for self-regulated learning.

This pattern of use had 2 consequences. First, students with few goals who did not set new goals were prone to quit the app and transition to a working attitude. For these students, only using daily reflections hindered their self-regulated learning. Second, without structural moments of deeper reflection, students potentially stay stuck in superficial learning, ultimately not reaching their learning goals [37].

#### Lessons for Designing Reflection Apps for Use in Clinical Placement Learning

The key finding in this unintended pattern of use is that supporting students through the self-regulated learning phases does not require in-app, phase-specific activities. In our case, supporting students in remembering and monitoring their learning goals seemed to already enable self-regulated learning. This highlights that students can go through these phases *outside* of the app, and that self-regulated learning is not a strictly linear process [43,44]. It is unsurprising that, given the nature of clinical placement learning, learning cannot be completely scripted. Thus, a reflection app for clinical placement learning should focus on enabling self-regulated learning and avoid a rigid process of in-app activities.

When it comes to enabling self-regulated learning, our findings show the importance of removing practical barriers. One barrier was forgetfulness. Students wanted reminders for their daily reflections, with some quitting the app after a period of inactivity. Similarly, students could be reminded to review their goals to maintain an intentional learning attitude. Thus, the intensive nature of clinical placement requires reflection apps to reduce the cognitive load of managing one’s learning process.

Another barrier ties to our weekly reflection—ease of access. Students who did write weekly reflections found them to be useful but lacked triggers to reflect more often. Those who did not use them were interested yet lacked a trigger to try them. One could address this by facilitating students to write deeper reflections when they realize they want to during the daily reflection. One unimplemented feature was to have the free writing field be interchangeable with reflection frameworks (eg, ALACT by Korthagen et al [45]). Future research into such features is needed to understand how a reflection app can facilitate students’ shifting reflection needs.

### Students’ Changing Needs Throughout Clinical Placement

A final key finding was the decline in app use, with nearly all students no longer using the app after the halfway point (10 wk). Interestingly, other studies with similar app interventions do not report this decline in use while having similar, mostly positive experiences.

One reason could be that those studies featured shorter clinical placement periods, such as 3 weeks [27,28], 6 weeks [20,46,47], or 8 weeks [12]. Yet, some studies that spanned a similar period of time do not report a decline in use [13,31]. From what we can tell, the difference there lies in whether using the tool was a required part of clinical placement learning. Shajani et al [13] digitized an existing, required reflection exercise, and Slade and Downer [31] measured students’ perceptions of electronic portfolios already being used in a curriculum. Thus, one reason for our dropout rate could be that it was optional to use.

Looking at the interview and focus group findings, we believe the decline is partially due to students experiencing a transitional phase during clinical placement. Students valued the structure provided by the app in the *initial, tumultuous phase*. Students’ reflections and goals reflect this as they focused mostly on learning protocols, how the team operates, and building rapport with patients. Similarly, this is when they learned clinical skills through hands-on experience. Thus, the urgency of the learning goals was high—“I need to function within the team!”—and one’s day-to-day growth was more palpable—“I am doing things I could not do last week!”

As this phase ended, *how* students used the app influenced further use. Students whose learning goals focused on clinical skills lacked urgency to further monitor their progress and quit the app. These students often did not manage their learning goals, whereas students with a large set of goals or who used a journaling approach stayed active longer. This indicates certain types of use are more conducive to self-regulated learning and should be advised.

Study coaches remarked that it is harder to get students to critically reflect on their learning process in the second half of the clinical placement. This is seen in how students lost awareness of their learning process and stopped reflecting after app use faded. Moreover, some students missed the oversight and, in hindsight, felt like they would have benefited from further use. This indicates that the work-oriented attitude and its unintentional learning process can hinder self-regulated learning.

The interviewees described clinical placement as being more conducive to learning the starting-phase skills. Most colleagues were willing and capable of helping students reach these goals, for example, through modeling procedures, giving clinical feedback, or explaining protocols. Contrastingly, and in line with other research [15,17], SEC—such as giving feedback or conflict management—were harder to discuss and thus found less uptake by students. Additionally, such skills bear less urgency and are more abstract than, for example, learning a new procedure. Thus, clinical placement as a learning environment and the nature of learning SEC within such an environment also influenced whether students were able to use it for that purpose.

Given the above finding, we believe other intervention components are needed to prepare students for practicing SEC during clinical placement. The app’s goal-setting and reflective structure then enables further learner agency and self-regulated learning of SEC. For example, only the few students in our field trial with clear goals regarding SEC managed to work on them. The study coaches acknowledged this, saying students who start with clearer goals are more likely to maintain a learning attitude. Simulation learning could help prepare students to practice with SEC before clinical placement [3,19,48,49]. Subsequently, SEC becomes more recognizable and their urgency clearer, enabling students to set concrete goals and tasks.

Overall, the app’s structure fulfills students’ early clinical placement learning needs. However, the app’s current design and how it was introduced to students do not support the more intentional approach needed for prolonged use. Looking at a recent similar study [12], we believe a stronger integration with the coaching process could help stimulate a more intentional approach (eg, revising or setting new learning goals). Thus, the design and implementation of a reflection app like ours needs to consider these changing needs to reach its intervention goals.

### Limitations

Two data collection issues occurred during the field trial. First, we observed low response rates to our first survey. Students struggled to keep up with our intended pace for surveys, as they were overwhelmed by the first weeks of clinical placement. Moreover, the surveys were the only part of the field trial that did not benefit their own learning process. We took two measures to address the low response rates:

1. Financial compensation: Students would receive a gift coupon of €10 (US $11.40) if they filled in at least 2 of the 3 surveys. Although this could introduce response bias, we aimed to mitigate this by framing the incentive as fair compensation for their time spent on the surveys. Additionally, research shows incentives to be an effective tool to increase response rates with no clear indication of lower response quality [50].
2. More time between surveys: We slowed down the pace of surveys to allow students more time to fill them in.

The second issue was technical and required us to stop the trial in the 13th week. As a result, we could not send out the third qualitative survey nor gather further log data. At that time, there were only 2 active users left, meaning comparatively little log data were lost. Similarly, the third survey would have had a low response rate. Nevertheless, the technical issues did bar us from measuring how students used the reflections to prepare for their clinical placement assessment, a question which could be addressed in future research.

The open setup of the study made it harder for us to evaluate our intended reflective process. We did explain the idea behind the types of reflection and the self-regulated learning cycle as a potential way to use the app. However, we did not require this pattern of use due to the exploratory nature of the study; we were interested in how they themselves would use it. Thus, although our approach led to insights in the variety of ways students used the app in their learning process, our study lacked insight into the potential effect of our intended reflective process.

Similarly, the small sample size, lack of a control group, and design of our study limited our capability to measure the effect of the app on student learning and SEC development. Nevertheless, this means our findings should be interpreted with caution and for their descriptive value of students’ experiences with the app and its role in their learning process. Future experimental research should look into the effect of reflection apps on SEC development during clinical placement. Finally, our small sample size and focus on one specific clinical placement in one specific nursing program limit the generalizability of our findings. We addressed this inherent limitation by supporting transferability of our findings to different contexts. For example, transparency in the design of the app and its underlying guidelines helps other researchers understand the intent behind design choices and gives context to our findings. Similarly, the mixed methods approach provides a rich description of the context, which further supports other researchers’ understanding of the relevance of our findings to their context. In doing so, we aim to support future research on this topic.

### Conclusions and Implications for Practice

The study’s exploratory and naturalistic nature allowed us to follow students’ developments throughout the clinical placement and the role of the app within it. The findings show that supporting students in structuring and monitoring their clinical placement learning helps them during the initial phase of the clinical placement. Although limited, our study indicates that capturing experiences as opposed to logging clinical procedures is key in using reflection for SEC development purposes. Concurrently, we see that the social norms of the workplace impact students’ SEC development, indicating interventions should address this in their design.

Future research should investigate how educational interventions can adapt to students’ changing attitudes toward learning as the clinical placement progresses. Additionally, for SEC development specifically, research into multicomponent intervention designs is needed to help students practice these competencies during clinical placement. Our findings can inform designers and nursing educators of the complexities in designing for nursing students’ SEC development during clinical placement.

## Supplementary material

10.2196/91083 Multimedia Appendix 1 Qualitative survey.

10.2196/91083 Multimedia Appendix 2 Interview schema.

10.2196/91083 Multimedia Appendix 3 Coach focus group verbal consent and focus group topic list.

10.2196/91083 Multimedia Appendix 4 Demographic data.

## Abbreviations

SEC: socioemotional competencies
