# Supplementary material for: Exploring the Potential for Smartphone-Based Reflection to Support Socioemotional Competence Development in Student Nurses’ Clinical Placement: Mixed Methods Field Trial
Source: JMIR Nurs. 2026 Aug 5;9:e91083. doi: 10.2196/91083 (PMC13441119; doi:10.2196/91083)
Supplement: Supplementary file 1 [file nursing-v9-e91083-s001.docx]

1. To what extent do the questions you answer after a shift (daily reflections) help you reflect on what you are learning during your internship?
   a. Which questions and/or functions do you find helpful in this regard, and what makes them helpful to you?
2. To what extent do the questions you answer at the end of each week (weekly reflections) help you reflect on what you are learning during your internship?
   a. Which questions and/or functions do you find helpful in this regard and what makes them helpful to you?
3. To what extent do you think the time it takes to keep track of your learning goals (tasks, daily reflections, weekly reflections) is worth it in relation to the insights it provides?
4. To what extent does the overview provided by the app (graph, collected tips and tops) help you gain insights into how you are doing with your learning goals?
   a. What do you do with the insights you gain from this?
