# Supplementary material for: Exploring the Potential for Smartphone-Based Reflection to Support Socioemotional Competence Development in Student Nurses’ Clinical Placement: Mixed Methods Field Trial
Source: JMIR Nurs. 2026 Aug 5;9:e91083. doi: 10.2196/91083 (PMC13441119; doi:10.2196/91083)
Supplement: Supplementary file 2 [file nursing-v9-e91083-s002.docx]

| Theme | Dimension | Indicator | | Keywords | | Goal/questions |
| --- | --- | --- | --- | --- | --- | --- |
| Learning context | Student factors | Prior knowledge | | Clinical placement experience, prior education (e.g. vocational) | | To gain insight into their attitude during the clinical placement, how they perceived their chances of passing, and how they look back on the experience in general. |
|  |  | Affect | | Motivation, contentess with clinical placement type/location | |  |
|  |  | Learning | | Self-efficacy, learner agency | |  |
|  | Clinical placement | Scaffolding | | Existing structure, alone or with other students, having to do shifts alone | | To what extent did the student have to build a learning context, and how was the clinical placement a challenge in that regard? Was there room to learn, or was it mainly about survival? |
|  |  | Learning environment | | Relationship with workplace supervisor and immediate colleagues, degree of feeling supported in the learning process. | |  |
| Learning process | Activities/content | Intention | | Proactive, reactive, passive  Competence/learning outcome-oriented, ‘go with the flow’-oriented, counting down the days or eager to learn. | | How do they approach learning? What have they emphasized and what not? Have they grown/changed in this regard? |
|  |  | Topic | | Clinical, interprofessional, personal skills | |  |
|  | Regulation/strategy | Forethought | | Tasks and learning goals, ad hoc or structured | | How do they reflect on their services, learning experiences, etc.? How do they incorporate this into their learning objectives, services, and attitude toward the learning context? How aware are they of their internship as a learning environment for their competencies? Do they demonstrate higher-order thinking in this regard? |
|  |  | Performance | | Approach (see also: intention), asking for help, defending (learning) needs, sense of control | |  |
|  |  | Self-reflection | | Self-evaluation, depth, follow-up meaning | |  |
| Relation tot the app | Learning context | Fit | | Obstacles, functionalities, time, depth, support | | How was the app used within the learning context, what influenced this, and why? |
|  |  | Value | | Structure, mindset, attitude towards learning, self-efficacy | |  |
|  | Learning process | Activities | | Topics and learning goals, tasks. | | What role did the app play in the student's learning process and why? How did it come about and what was useful and what was not? |
|  |  | Regulation | | Self-monitoring, interventions, help-seeking. | |  |
| Can you tell me something about how you dealt with setting learning goals during your internship? And how did you then work on those learning goals? How did you initially incorporate the app into that? | | | What role did reflection play during your internship? Did you also do this outside of the app? If so, how? You mentioned that you mainly used the daily reflections. How did this help you? What did you gain from it? Can you give an example? | | How do you look back on what you learned during this internship? What role do you think you played in that? What was the role of the app in that, or how did you see the app playing a role in that? | |
