# Supplementary material for: Exploring the Potential for Smartphone-Based Reflection to Support Socioemotional Competence Development in Student Nurses’ Clinical Placement: Mixed Methods Field Trial
Source: JMIR Nurs. 2026 Aug 5;9:e91083. doi: 10.2196/91083 (PMC13441119; doi:10.2196/91083)
Supplement: Supplementary file 3 [file nursing-v9-e91083-s003.docx]

# Verbal consent script

Good morning, everyone,

First of all, thank you for agreeing to participate in this focus group. Before we begin, I would like to obtain your informed consent. By giving your consent, you consent that this session will be recorded and that the anonymized data will be used within the context of doctoral research. For example, in a publication. Finally, you acknowledge that you are participating voluntarily and can therefore withdraw from the focus group at any time.

Do you consent to this?

# Topic list

| Topic (+ introduction) | Question |
| --- | --- |
| General | Years of experience coaching this clinical placement module (PLP1) |
| Changing attitude of the student  For the majority of participating students, we saw a clear decline in the use of the app around the time of the halfway evaluation. During the interviews, it became clear that many students felt they needed the app less because they were becoming increasingly better at performing their daily tasks. As a result, their attitude shifted from ‘learning’ to ‘working’. The learning goals faded into the background. | To what extent do you recognize this transition based on your experience with PLP1? |
|  | Does this often also mean that the student truly has little or nothing left to learn? |
|  | How do you coach these students and/or what do you notice during follow-up education^1^? |
| Different groups of students  A small proportion of this group has internalized their learning objectives to such an extent that they are able to create or recognize learning opportunities. However, the majority of interviewees indicated that there was little left to learn (even though the interviews revealed that interprofessional skills or more continuous professional development-oriented skills still require attention). | To what extent do you recognize these two groups of students from your experience with PLP1? |
|  | What is your experience with students' attitudes toward continuous professional development learning goals during PLP1? Or the more interprofessional learning objectives? And does that attitude change during the internship? |
|  | To what extent do you see this reflected in the differences between the halfway evaluation and the final assessment? (little attention could mean little growth) |
| Difficulty of PLP1  Almost all of the students who participated indicated that the internship went well for them. To get a better picture of the average student, I wondered to what extent PLP1 is perceived as challenging by students. | In your experience, how challenging do students find PLP1? |
|  | Where do PLP1 students often get stuck, what do they find challenging about PLP1? How many (approximately) fail as a result? |
|  | How important are the continuous professional development learning goals and interprofessional learning goals for passing PLP1? |
