# Supplementary material for: Exploring the Potential for Smartphone-Based Reflection to Support Socioemotional Competence Development in Student Nurses’ Clinical Placement: Mixed Methods Field Trial
Source: JMIR Nurs. 2026 Aug 5;9:e91083. doi: 10.2196/91083 (PMC13441119; doi:10.2196/91083)
Supplement: Supplementary file 4 [file nursing-v9-e91083-s004.docx]

Participants’ average age 20 (SD 1.23) years, 87% female students. Table S1 shows the distribution of care organizations. Table S2 shows all demographic data.

Table S1: Distribution of types of care organizations.

| Type of Care | N |
| --- | --- |
| Hospital (Netherlands) | 1 |
| Hospital (Germany) | 6 |
| Home care | 3 |
| Nursing home | 2 |
| Mental healthcare | 3 |

Table S2: Demographic data per participant. Some age-data is missing as this was only asked at the semi-structured interviews, which not all students participated in.

| Participant # | Clinical placement type | Age | Sex |
| --- | --- | --- | --- |
| 907 | Home Care | 21 | F |
| 246 | Hospital (Germany) | 19 | F |
| 269 | Hospital | 21 | F |
| 202 | Nursing Home |  | F |
| 163 | Hospital (Germany) | 22 | F |
| 451 | Hospital (Germany) | 22 | F |
| 363 | Mental healthcare | 19 | F |
| 402 | Hospital (Germany) | 19 | F |
| 412 | Hospital (Germany) |  | F |
| 528 | Home Care | 18 | F |
| 960 | Home Care | 21 | M |
| 309 | Mental healthcare | 20 | M |
| 380 | Mental healthcare |  | F |
| 926 | Hospital (Germany) |  | F |
| 664 | Nursing Home | 21 | F |
